# Application of Solution Blow Spinning for Rapid Fabrication of Gelatin/Nylon 66 Nanofibrous Film

**DOI:** 10.3390/foods10102339

**Published:** 2021-09-30

**Authors:** Zhichao Yang, Chaoyi Shen, Yucheng Zou, Di Wu, Hui Zhang, Kunsong Chen

**Affiliations:** 1 Zhejiang Provincial Key Laboratory of Horticultural Plant Integrative Biology, College of Agriculture & Biotechnology, Zhejiang University, Hangzhou 310058, China; 22016147@zju.edu.cn (Z.Y.); scyzju@zju.edu.cn (C.S.); akun@zju.edu.cn (K.C.); 2 The State Agriculture Ministry Laboratory of Horticultural Plant Growth, Development and Quality Improvement, College of Agriculture & Biotechnology, Zhejiang University, Hangzhou 310058, China; 3 College of Biosystems Engineering and Food Science, Zhejiang University, Hangzhou 310058, China; yuczou@zju.edu.cn (Y.Z.); hubert0513@zju.edu.cn (H.Z.); 4 Zhongyuan Institute, Zhejiang University, Zhengzhou 450000, China

**Keywords:** solution blow spinning, gelatin, nylon 66, modification, rapid fabrication

## Abstract

Gelatin (GA) is a natural protein widely used in food packaging, but its fabricated fibrous film has the defects of a high tendency to swell and inferior mechanical properties. In this work, a novel spinning technique, solution blow spinning (SBS), was used for the rapid fabrication of nanofiber materials; meanwhile, nylon 66 (PA66) was used to improve the mechanical properties and the ability to resist dissolution of gelatin films. Morphology observations show that GA/PA66 composite films had nano-diameter from 172.3 to 322.1 nm. Fourier transform infrared spectroscopy and X-ray indicate that GA and PA66 had strong interaction by hydrogen bonding. Mechanical tests show the elongation at break of the composite film increased substantially from 7.98% to 30.36%, and the tensile strength of the composite film increased from 0.03 MPa up to 1.42 MPa, which indicate that the composite films had the highest mechanical strength. Water vapor permeability analysis shows lower water vapor permeability of 9.93 g mm/m^2^ h kPa, indicates that GA/PA66 film’s water vapor barrier performance was improved. Solvent resistance analysis indicates that PA66 could effectively improve the ability of GA to resist dissolution. This work indicates that SBS has great promise for rapid preparation of nanofibrous film for food packaging, and PA66 can be applied to the modification of gelatin film.

## 1. Introduction

Solution blow spinning (SBS) is a rising preparation technique to produce polymer films with ultrafine fibers and high specific surface area [1]. A regular SBS apparatus consists of a high velocity air source, a syringe pump, a concentric nozzle system, and a collector [2]. The polymer solution in the inner nozzle is elongated into fine fiber by the surrounding high velocity airflow in the outer nozzle. Moreover, because SBS uses high velocity airflow to form fibers, there is no requirement for the conductivity of the solution compared to electrospinning. As the solvent rapidly evaporates in the process of moving towards the collector, ultrafine fibers deposited and formed a nanofibrous film on the collector [3]. The SBS has served a wide range of purposes that include biosensors, the aerospace industry, impurity removers, and wearable electronics [4]. The development of nanofiber films for food packaging using SBS, however, has undergone little research. For the present, the utilization of nanofiber film in food packaging is primarily manufactured by the electrospinning method [5,6,7,8,9]. Nonetheless, previous works show that the fiber production efficiency of SBS could be much higher than electrospinning. Tandon et al. [10] found that SBS was three times more efficient in fiber production than electrospinning; and in the study of Sett et al. [11], the fiber productivity of SBS was even up to 30 times higher than that of electrospinning. Shen et al. [12] also found that the SBS process could reach a rapid feed rate of 3 mL/h, while the feed rate of the electrospinning is generally between 0.1 mL/h and 0.6 mL/h. In this regard, the SBS technology has demonstrated its promising potential for the fast production of large area nanofiber film for food packaging.

Gelatin (GA) is a single-stranded protein obtained by hydrolysis of collagen, with favorable biocompatibility, biodegradability and nontoxicity, and has been utilized extensively in the food, pharmaceutical and photographic industries [13,14,15]. However, due to the solubility in aqueous solution and poor mechanical properties, gelatin nanofibers usually require modification by chemical cross-linking agents [16,17]. Bigi et al. [18] investigated the mechanical and swelling properties of glutaraldehyde (GTA) crosslinked gelatin films, and found that the use of GTA allowed to regulate the physical-chemical properties of gelatin films. But the residue and cytotoxicity of chemical agents have limited its application in the food field [19].

Various studies have shown that many synthetic polymers such as poly(caprolactone) (PCL), poly(glycolic acid) (PLA), poly(lactic-co-glycolic acid) (PLGA) can be used with natural polymers to produce polymer composites with improved performance [20,21,22,23,24]. Among these synthetic polymers, nylon 66 (polyamide 66, PA66) is a multifunctional synthetic thermoplastic polymer broadly used in textiles, parachutes, biomedical fields, and functional materials [25]. It was reported that PA66 has been successfully used to modify epoxy resins to achieve tensile toughness enhancement [26], however there has been limited work related to the use of PA66 for the modification of natural polymers, especially gelatin. Owing to its anti-abrasion resistance, high mechanical strength, light weight, relatively low cost and, very importantly, biological safety [27,28,29,30,31], PA66 can be used as a potential modification material for gelatin.

In this work, SBS was used to rapidly fabricate GA/PA66 composite nanofiber films to address the need for food packaging materials to be prepared on a large scale. The feasibility of PA66 as the modifier to enhance comprehensive performance of gelatin in mechanical properties and the ability to resist dissolution was evaluated. The morphology, diameter distribution, molecular interaction, crystal structures, thermal stability, mechanical strength, water contact angle (WCA) and water vapor permeability (WVP) of the GA, GA/PA66 and PA66 films were characterized. In addition, the prospects of SBS for rapid preparation of food packaging are discussed.

## 2. Materials and Methods

### 2.1. Chemicals

Gelatin (GA, Bloom 250 g) was obtained from Aladdin, Inc. (Shanghai, China). Nylon 66 (PA66, pellets) was obtained from Sigma Aldrich (St. Louis, MO, USA). 88% (*v*/*v*) Formic acid and glacial acetic acid (analytical reagent grade) were acquired from Sinopharm Chemical Reagent Co., Ltd. (Shanghai, China).

### 2.2. Spinning Solution

GA (12% *w*/*v*) solutions were obtained through dissolving 1.2 g GA in 10 mL glacial acetic acid after sufficient stirring overnight. PA66 (12% *w*/*v*) solution was obtained through adding 1.2 g PA66 pellets into 10 mL formic acid with stirring overnight. Blend solutions at a final concentration of 12% (*w*/*v*) with gelatin/PA66 weight ratios of 1/2, 1/1 and 2/1 were obtained through dissolving gelatin and PA66 in glacial acetic acid and formic acid after stirring overnight. The solution was stirred for 6 h and ultrasonic treated for 30 min before the SBS process.

### 2.3. Solution Blow Spinning (SBS) Process

The JNS-SBS-01 SBS apparatus (Janus New-Materials Co., LTD, Nanjing, China) was employed to fabricate the nanofibrous films. Solutions were added into a syringe, and the feed rate of syringe pump was 3 mL/h. The distance between nozzle and reticular collector was 20 cm. An air pressure of 0.06 MPa was used to provide a stable shear force. The SBS process was performed at 30 °C with a relative humidity of approximately 50%. Obtained films were GA film, PA66 film and gelatin/PA66 (2:1, 1:1, 1:2) films, respectively.

### 2.4. Fiber Morphology

The nanofiber morphology of the films was observed by FE-SEM (GeminiSEM 300, ZEISS, Oberkochen, Germany). The average diameters of nanofiber and their distributions were obtained through randomly measuring 100 fibers with Nano Measurer 1.2 (Fudan University, Shanghai, China).

### 2.5. Fourier Transform Infrared (FTIR) Analysis

FTIR analysis was carried out by method of KBr pellet to determine interactions among the components. The FTIR spectrum was obtained by using a Nicolet 170-SX instrument (Thermo Nicolet Ltd., Waltham, MA, USA). The wavenumber range was 4000–400 cm^−1^ and an average of 32 scans at 4 cm^−1^ were performed for each measurement.

### 2.6. X-ray Diffraction (XRD) Analysis

The crystal structure of the nanofiber films can be examined through XRD. XRD analysis was performed through an X’Pert Pro diffractometer (PA Analytical B.V., Eindhoven, The Netherlands) with the conditions at 40 kV, 35 mA. The diffraction range was 5–90° (2θ) and the rate of scanning was 2° min^−1^.

### 2.7. Thermal Analysis

DSC was performed through a TA Q200 instrument (TA Instruments, Newcastle, DE, USA) with the heating range of 20 °C to 300 °C. TGA was performed through a TA Q500 instruments (TA Instruments, Newcastle, DE, USA) with the heating range of 50 to 600 °C. The rate of heating for DSC and TGA was 10 °C min^−1^.

### 2.8. Mechanical Strength

The mechanical strength of the nanofibrous films was measured through a mechanical testing instrument (Instron 5944, Norwood, MA, USA) quipped with a loadcell of 10 N used at ambient temperature. The force rate was 1 mm min^−1^. The films were tailored into strips with a thickness of 0.1 mm.

### 2.9. Water Contact Angle (WCA)

A OCA20 device (Data Physics Co., Ltd., Bad Vilbel, Germany) was employed to investigate the WCA of the nanofibrous films through the sessile drop method [32]. The nanofibrous films were fixed on the object slides, and then a droplet of distilled water (3.5 μL) was deposited on the nanofibrous films. The water drops at 0 and 3 s were both recorded and averaged to determine the values of the WCA. The WCA value was computed from three positions on the films [33].

### 2.10. Water Vapor Permeability (WVP)

The ASTM E96 gravimetric method was used to calculate the WVP values. Fibrous film was fixed on top of a permeation cup filled with distilled water and kept in a desiccator loaded with dry silica gel. Following the attainment of steady state (approximately 1 h), the permeation cups were weighed every 12 h during a 5-day period. The WVP was obtained according to the following Equation [34]:

(1) $$\mathrm{WVP}=\frac{\Delta M\times d}{\Delta t\times A\times \Delta p}$$

where Δ*M*/Δ*t* is the weight of water loss per unit time (g h^−1^), *d* is the film thickness (mm), *A* is the area of the film exposed to moisture (m^2^), and Δ*p* is the water vapor pressure difference crossing the film (3.1671 kPa at 25 °C), respectively.

### 2.11. Solvent Resistance

The GA, PA66, and GA/PA66 films were tailored into round sheets and immersed in 1 × PBS for 24 h at room temperature, respectively. After drying at room temperature, the morphology of the samples was observed using field-emission scanning microscopy.

### 2.12. Statistical Analysis

All data were prepared in triplicate and expressed as the mean values ± standard deviation (SD). One-way ANOVA was performed using SPSS 19.0 statistical software (IBM Corp., Armonk, NY, USA) followed by Duncan’s multiple comparison test; *p* < 0.05 had statistical significance.

## 3. Results and Discussion

### 3.1. Morphologies of Nanofibrous Films

**Figure 1:**
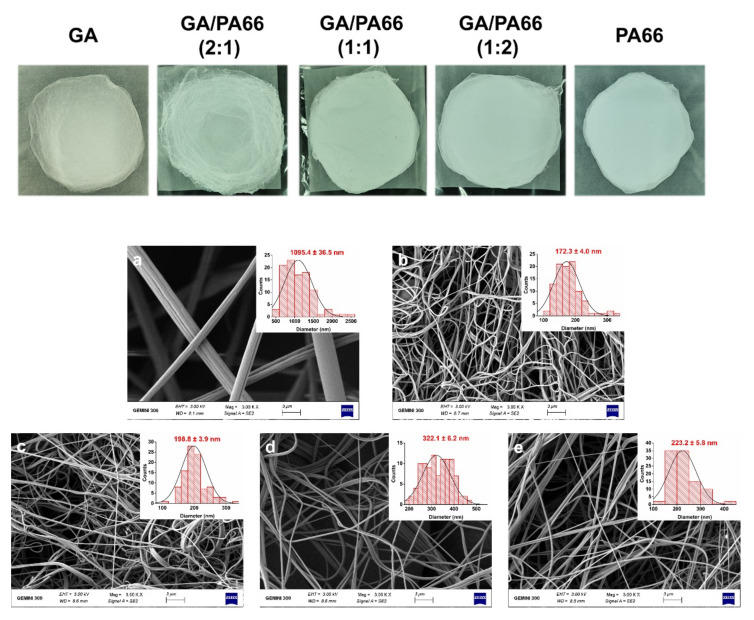
Pictures and scanning electron microscopy (SEM) images of the solution blowing spun gelatin (GA) (**a**), GA/PA66 (2:1) (**b**), GA/PA66 (1:1) (**c**), GA/PA66 (1:2) (**d**), PA66 (**e**) nanofibers.

Figure 1 shows the pictures of each film and the morphologies and diameter distributions of the solution blowing spun GA, PA66 films and GA/PA66 films with various weight ratios (2/1, 1/1 and 1/2 *w*/*w*). The average diameter of pure GA fibers was over 1000.0 nm, and the average diameter of pure PA66 fibers was 223.2 nm; with the weight ratio of PA66 increased from 1:2 to 2:1, the diameters increased from 172.3 nm to 322.1 nm. It can be observed that the addition of PA66 resulted in a significant reduction in fiber diameter, thus creating a denser network structure, which could improve the water vapor barrier performance. This was probably due to the fact that the addition of PA66 reduced the viscosity of the solution and promoted the elongation of the nanofibers, resulting in a thinner diameter [12]. In a similar study of modification of gelatin by Meng et al. [22], they found the average diameter of GA/poly (lactic-co-glycolic acid) (PLGA) nanofibers also decreased with the addition of PLGA. Moreover, the pure GA fibers were straight, while after the addition of PA66, the GA/PA66 composite fibers showed a curly appearance. In addition, the usage of pure gelatin solution for spinning resulted in a large number of droplets, so the film had hard lumps, leading to low solution utilization; while after the introduction of PA66, the droplets were significantly reduced and the phenomenon of hard lump formation disappeared, making the solution utilization improve. Therefore, this indicates that the addition of PA66 led to the improvement of GA spinnability during the SBS fabrication of nanofiber films. Like other spinnable polymers, nylon may increase the viscoelasticity of the gelatin-containing precursor solution, allowing for easier fiber formation [35].

### 3.2. FTIR Spectra Analysis

**Figure 2:**
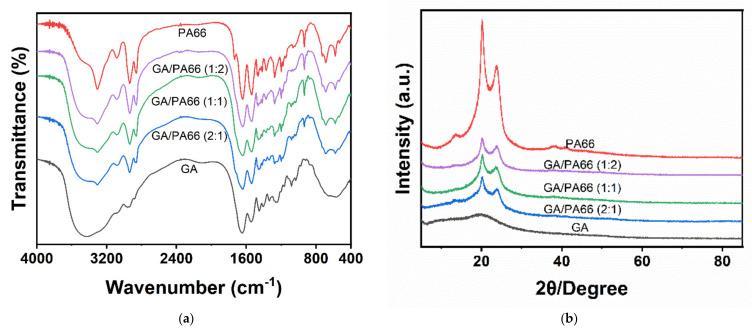
(**a**) Fourier transform infrared (FTIR) spectra and (**b**) X-ray diffraction (XRD) patterns of the GA, GA/PA66 (2:1), GA/PA66 (1:1), GA/PA66 (1:2), PA66 nanofibers.

Figure 2a shows the FTIR spectra of the GA, PA66 films and GA/PA66 films with various weight ratios. For the GA film, the broad stretching band at 3000 to 3750 cm^−1^ (amide A) was related to N–H stretching vibrations and O–H, and three characteristic peaks of GA were observed at around 1647 cm^−1^ (amide I) corresponding to C=O and C–N stretching vibrations, 1542 cm^−1^ (amide II) corresponding to N–H bending and C–H stretching vibration, and 1259 cm^−1^ (amide III) [9,36]. For PA66 film, the adsorption peak appearing at 3305 cm^−1^ belonged to the N–H stretching band of amine group and the peaks at 1641 cm^−1^ and 1540 cm^−1^ [37]; and the characteristic peak at 2934 cm^−1^ corresponding to the stretching vibration of C–H also confirmed solid evidence of the existence of PA66 [38].

The absence of peak splitting in all mixed nanofibers indicates that gelatin and PA66 were uniformly dispersed in the fibers. Overall, the GA/PA66 composite films exhibited similar major peaks, but with different amplitudes. The characteristic peaks of PA66 at around 3305 and 2934 cm^−1^, and three characteristic peaks of at around 1647, 1542 and 1259 cm^−1^ belonging to GA, were observed in the GA/PA66 composite films, indicating that the introduction of PA66 did not disrupt the structure of GA and the homogeneous mixing in the GA/PA66 composite film. In addition, it can be observed that the peak around 3436 cm^−1^ in GA shifted to a higher band at about 3305 cm^−1^. The lower intensity of the peak in the range 3125 cm^−1^ and 3400 cm^−1^ may be attributed to the interaction between the O–H and N–H groups of gelatin and PA66 molecules resulting in more intermolecular or intramolecular hydrogen bonding [20]. This result may also explain the phenomenon of improved GA spinnability mentioned above.

### 3.3. XRD Analysis

Figure 2b shows the XRD results of the GA, PA66 films and GA/PA66 films with various weight ratios. PA66 has two obvious diffraction peaks at around 2θ = 20.1° ascribed to the interchain hydrogen bond plane of the amide group [29] and 2θ = 23.7°, which is similar to the results of Noguchi’s study [39]; whereas gelatin had a broad diffraction peak at 2θ = 19.2°, suggesting a low crystallinity of GA. For the GA/PA66 composite films, two diffraction peaks appeared between 20° and 25°, which were corresponding to the two characteristic peaks (α1 and α2) of the α-crystalline form of PA66 [38]. It was noticeable that the intensity of the peaks of the GA/PA66 composite film was enhanced compared to the pure GA film, suggesting that the crystallization of the GA/PA66 composite films was advanced with the addition of PA66.

### 3.4. Thermal Analysis

**Figure 3:**
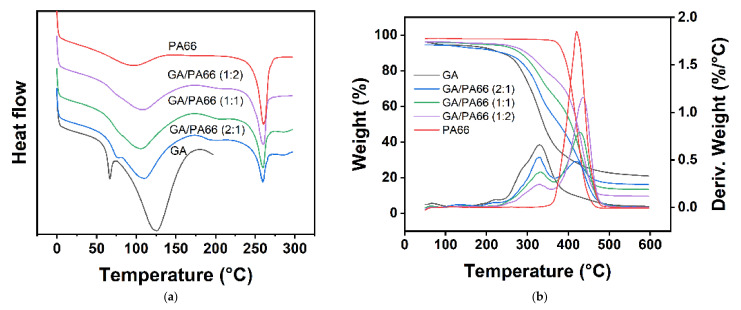
(**a**) Differential scanning calorimetry (DSC) and (**b**) thermogravimetric analysis (TGA) curves of the GA, GA/PA66 (2:1), GA/PA66 (1:1), GA/PA66 (1:2), PA66 nanofibers.

Figure 3a shows the DSC curves of the GA, PA66 films and GA/PA66 films with various weight ratios. The melting temperature (T_m_) and melting enthalpy (ΔH_m_) are summarized in (Table 1). The T_m_ of the pure GA and PA66 films were 126.50 °C and 260.43 °C, respectively. It should be noticed that the T_m_ and ΔH_m_ of the GA/PA66 composite films decreased with the addition of PA66. This might be because the GA nucleation was promoted to generate crystalline regions after the introduction of PA66.

Figure 3b shows the TGA curves of the nanofibrous films, and the details were provided in (Table 1). The results indicate that there was a weight loss in the period from 50 to 260 °C, which was related to the vaporization of moisture [40]. After approximately 300 °C, a higher loss of nanofiber films resulting from polymer decomposition was recorded. The decomposition of GA occurs at about 300 °C, while the decomposition of PA66 at around 420 °C was observed. As the temperature hits 600 °C or higher, the mass of the films tended to be stable, indicating that the decomposition of the films was complete. At the completion of the TGA, the residual weights of the pure GA and PA66 films were 21.04% and 2.95%, respectively. The residual weights of the GA/PA66 composite films were 16.20%, 13.50% and 9.70%, corresponding to weight ratios of 2/1, 1/1 and 1/2, which were negatively correlated with the weight of PA66. The above results revealed that the residual amount of the composite films was reduced, while the rate of decomposition and the maximum decomposition temperature increased.

### 3.5. Mechanical Properties Analysis

**Figure 4:**
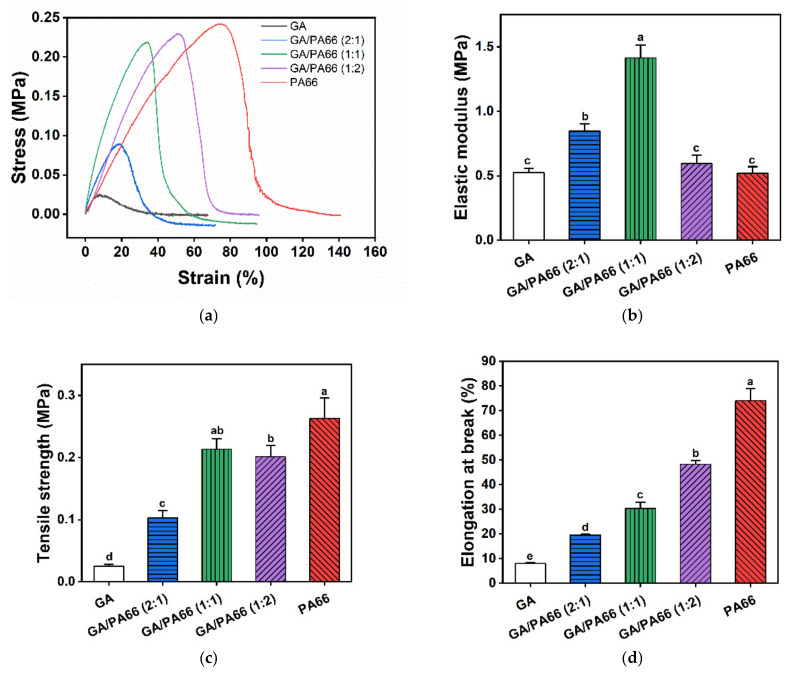
Mechanical properties of the nanofibrous films: (**a**) stress-strain curves; (**b**) elastic modulus; (**c**) elongation at break; (**d**) tensile strength. Values denoted with different letters (a–e) are significantly different (*p* < 0.05), where a is the highest value.

Figure 4 shows the mechanical properties of the GA, PA66 films and GA/PA66 films with various weight ratios. The elastic modulus, elongation at break, and tensile strength of the GA film were 0.52 MPa, 7.98%, and 0.03 MPa, respectively. This result implied that the GA film was a material with poor ductility and mechanical strength. After blending with PA66, the elongation at break of the GA film increased substantially from 7.98% to 30.36% at the weight ratio of 1:1 (GA/PA66), and the tensile strength of the GA film were considerably enhanced from 0.03 MPa up to 1.42 MPa, which was 48 times higher, but there was a certain range of differences compared with pure PA66 nanofiber film. This might be due to the fact that the reduction of the diameter of nanofibers led to an increase in the density of inter-fiber alignment, and the uniformity of the nanofiber film might be improved [41]. In a similar study of Zhang et al. [21], they found that the mechanical properties of the GA films was also improved with the addition of poly(ε-caprolactone). In addition, the elongation at break of GA/PA66 was significantly improved compared to the pure GA film. These results indicate that the composite films had higher mechanical strength due to the addition of PA66.

### 3.6. Water Contact Angles Analysis

**Figure 5:**
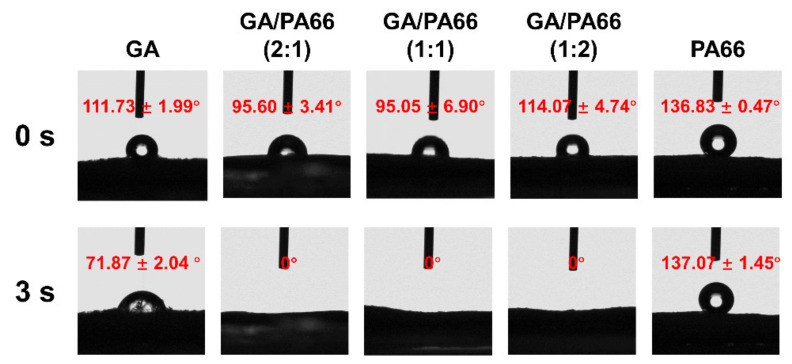
Water contact angles of the films at 0 s and 3 s (equilibration time).

Figure 5 shows the WCAs of the films at the equilibration times of 0 s and 3 s. The WCA of the GA film was 111.73° at 0 s and 71.87° at 3 s, which implied the GA film had a comparatively hydrophilic surface. This was because GA was a hydrophilic material [42]. The WCA of the PA66 film was 136.83° at 0 s and 137.07° at 3 s, which meant the PA66 film had a comparatively hydrophobic surface. However, the hydrophilicity of GA/PA66 films was significantly increased since the composite films had a super hydrophilic surface of 0° at both 0 s and 3 s. Similar results were reported by Cao et al. [43], who found that compared to pure polyethersulfone film, the hydrophilicity of the polyethersulfone/PA66 filtration film surface was improved. This may be related to the fact that a considerable number of hydrophilic groups existed in PA66 [44], and the homogeneous dispersion of gelatin in the mixing system, thus exposing the hydrophilic groups more sufficiently to the surface. The hydrophilic surfaces of materials have been considered as an important factor in increasing the antimicrobial agent loading and antimicrobial activity. Karam et al. [45] found that hydrophilic surfaces could load more antimicrobial agents (nisin) and had higher antimicrobial activity than hydrophobic surfaces. Therefore, the GA/PA66 composite film presents promising potential in loading high doses of antimicrobial agents for food packaging.

### 3.7. Water Vapor Permeability Analysis

**Figure 6:**
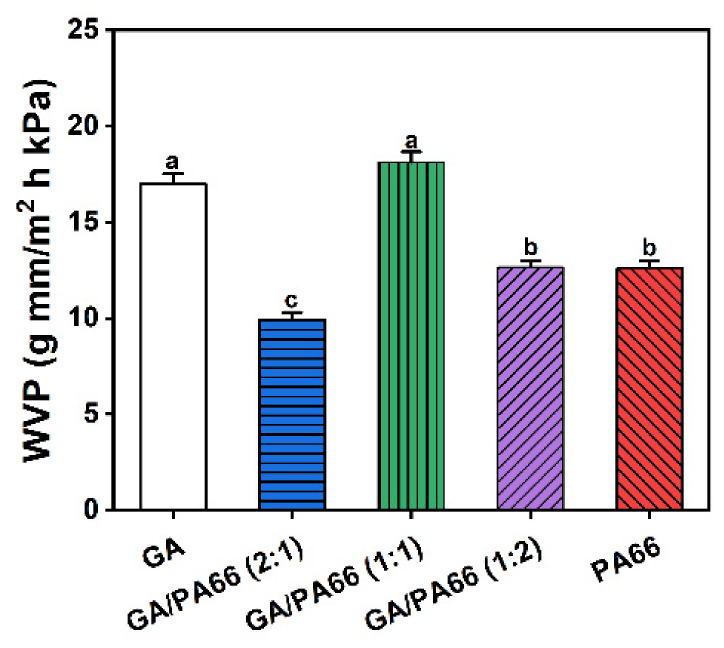
Water vapor permeability of the nanofibrous films. Values denoted with different letters (a–c) are significantly different (*p* < 0.05), where a is the highest value.

The rate of water vapor transmission was investigated to be proportional to the porosity of the nanofiber film due to the fiber diameter [46]. Figure 6 shows the WVP values of the GA, PA66 films and GA/PA66 films with various weight ratios. It is well acknowledged that WVP is an inevitable factor of packaging products, which is associated with the exchange of water with the food and the environment [34]. The WVP values of the pure GA and PA66 nanofibrous mat were 16.99 and 12.61 g mm/m^2^ h kPa, respectively. When mixing with PA66, the WVP decreased significantly at weight ratios of 2:1 and 1:2 (GA/PA66), and the WVP for the GA/PA66 composite film declined from 16.99 to 9.93 g mm/m^2^ h kPa at weight ratios of 2:1 (GA/PA66), indicating that the water vapor barrier performance was improved. This improved water vapor barrier performance could be attributed to a higher crystallization, which could function as a nucleating element, producing increased crystallinity and impermeable regions in the film [47]. In a similar study of the modification of gelatin by Mohammadi et al. [48], they also found that the WVP of GA/chitosan composite film significantly decreased after chitosan was added.

### 3.8. Solvent Resistance

**Figure 7:**
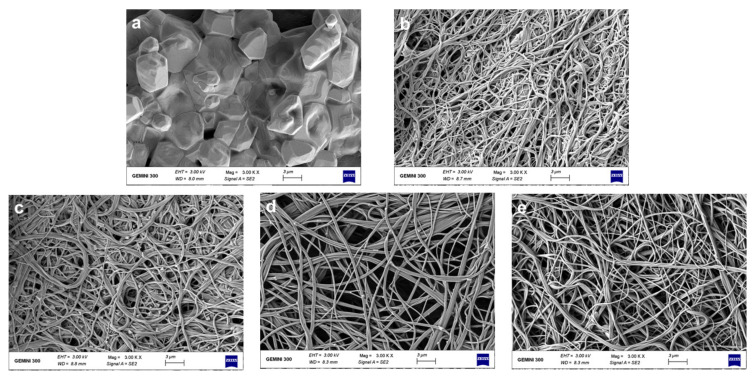
Morphologies of the GA (**a**), GA/PA66 (2:1) (**b**), GA/PA66 (1:1) (**c**), GA/PA66 (1:2) (**d**) and PA66 (**e**) films after immersed in 1 × phosphate-buffered saline (PBS) for 24 h.

Figure 7 shows the morphologies of the PA66 films and GA/PA66 films after immersing them in 1 × PBS for 24 h and drying. The pure gelatin film (Figure 7a) swelled in 1 × PBS solution, completely losing their fibrous structure and dried to form a transparent solid with a block-like structure, while the pure PA66 film (Figure 7e) remained intact in its fibrous form. With the addition of PA66, the fiber structure of the composite films (Figure 7b–d) was well maintained, indicating that the addition of PA66 could effectively improve the ability of GA to resist dissolution and thus maintained the various advantages brought by its nanometer size. The explanation for this could be that the PA66 network provided good support in an aqueous environment to maintain the basic structure of the gelatin. This intact fiber structure could improve the barrier performance of the fiber film in humid environments. The above results are consistent with the observation of Deng et al.’s work [16], which found that zein particles were distributed nicely in the gelatin network and helped preserve the compact structure by co-electrospinning. As reported by Ahammed et al. [49], the resistance to water of GA was also improved with the solubility in water decreased from 100% to 40% after the introduction of zein. The improved solvent resistance allows GA/PA66 composite films to be used for packaging of foods that generate water vapor due to respiration, such as fruits and vegetables.

## 4. Conclusions

In this work, GA/PA66 composite films were fabricated successfully by the SBS technique. The addition of PA66 resulted in a significant reduction in the diameter of the composite film. The SBS process mixed GA and PA66 into a homogeneous system by forming hydrogen bonding. Compared to the pure gelatin film, the GA/PA66 composite films had higher mechanical strength and the solvent resistance was improved. Overall, the comprehensive observation of GA/PA66 composite films indicated that PA66 can be used as a modified material for gelatin to improve the mechanical properties and solvent resistance, and SBS presents great promise for the rapid preparation of large area nanofiber film for food packaging.

## Figures and Tables

**Table 1:** Detailed data of the DSC and TGA thermograms for the nanofibrous films.

Sample	DSC Parameters				TGA Parameters		
	T_m_, _GA_ (°C)	ΔH_m_, _GA_ (J/g)	T_m_, _PA66_ (°C)	ΔH_m_, _PA66_ (J/g)	T_10wt%_ (°C) ^1^	T_max_ (°C) ^2^	W_red_ (%) ^3^
GA	126.50	145.10	/	/	236.70	329.59	21.04
GA/PA66 (2:1)	110.20	148.7	259.71	21.87	240.17	330.22	16.20
GA/PA66 (1:1)	106.22	115.3	259.81	28.74	282.64	427.51	13.50
GA/PA66 (1:2)	108.28	102.9	260.39	38.52	298.13	435.59	9.70
PA66	/	/	260.43	65.61	387.09	420.18	2.95

^1^ T_10wt%_ was the temperature at 10% mass loss. ^2^ T_max_ was the temperature at maximum weight loss rate. ^3^ W_red_ was the residual weight at 600 °C.

## Data Availability

Not applicable.
